# Multivariate CNN Model for Human Locomotion Activity Recognition with a Wearable Exoskeleton Robot

**DOI:** 10.3390/bioengineering10091082

**Published:** 2023-09-13

**Authors:** Chang-Sik Son, Won-Seok Kang

**Affiliations:** 1 Division of Intelligent Robot, Daegu Gyeongbuk Institute of Science & Technology (DGIST), Daegu 42988, Republic of Korea; changsikson@dgist.ac.kr; 2 Department of Biomedical Science, Graduate School, Kyungpook National University, Daegu 41944, Republic of Korea

**Keywords:** human activity recognition, wearable robot, single-head CNN, multi-head CNN, hyperparameter optimization, time series classification

## Abstract

This study introduces a novel convolutional neural network (CNN) architecture, encompassing both single and multi-head designs, developed to identify a user’s locomotion activity while using a wearable lower limb robot. Our research involved 500 healthy adult participants in an activities of daily living (ADL) space, conducted from 1 September to 30 November 2022. We collected prospective data to identify five locomotion activities (level ground walking, stair ascent/descent, and ramp ascent/descent) across three terrains: flat ground, staircase, and ramp. To evaluate the predictive capabilities of the proposed CNN architectures, we compared its performance with three other models: one CNN and two hybrid models (CNN-LSTM and LSTM-CNN). Experiments were conducted using multivariate signals of various types obtained from electromyograms (EMGs) and the wearable robot. Our results reveal that the deeper CNN architecture significantly surpasses the performance of the three competing models. The proposed model, leveraging encoder data such as hip angles and velocities, along with postural signals such as roll, pitch, and yaw from the wearable lower limb robot, achieved superior performance with an inference speed of 1.14 s. Specifically, the F-measure performance of the proposed model reached 96.17%, compared to 90.68% for DDLMI, 94.41% for DeepConvLSTM, and 95.57% for LSTM-CNN, respectively.

## 1. Introduction

Wearable exoskeleton robots have been developed to aid individuals in a range of activities, including carrying heavy objects, alleviating the burden of physically demanding tasks, and assisting in-patient rehabilitation. Studies have indicated that exoskeletons can substantially assist and lower metabolic costs during walking [1,2]. Numerous powered exoskeleton robots have facilitated the improvement of lower extremity movement deficits resulting from strokes [3,4,5] or injuries such as amputations [6,7] by applying assistive torques to the joints [8]. However, despite these successful applications, several challenges persist in developing safe and versatile control systems [9], including the identification of the wearer’s intended movement without external commands, and the autonomous transition between different activity-specific controllers.

One approach to identifying intended activity involves using a locomotor activity intent recognition framework [10,11]. This method is predominantly applied in medical rehabilitation, analyzing patients’ gait patterns to furnish clinicians with a quantitative overview of motor function behavior over extended durations, thus aiding objective treatment strategy applications [12]. For instance, due to postural instability and gait disturbances, Parkinson’s disease patients have an increased susceptibility to fall-related injuries [13,14]. Real-time movement monitoring can mitigate injury risks by promptly identifying fall hazards. Intent recognition technology augments current methods by pinpointing disease-specific predictors such as tremors and hyperkinesia [15,16], differentiating symptoms across varied motor activities. Accurately discerning an individual’s intended locomotion can also offer data that facilitate the adaptive control of assistive devices or wearable robots. Several studies have implemented activity intent recognition strategies by leveraging sensor fusion [10,11,17]. Specifically, [10] employed multiple sensors to monitor the internal state of the prosthesis (i.e., joint angles and angular velocities), as well as to gather information about user-environment interactions (i.e., forces and torques) to control the prosthesis for various activity modes (e.g., walking, standing, sitting). Trials with a unilateral amputee subject demonstrated that the Gaussian mixture model (GMM)-based intent recognition framework can identify user intent in real time and transition to the appropriate activity control. However, intent recognition in this study was reliant on handcrafted features extracted from the prosthesis signals, such as mean and standard deviation. This raises a challenge as temporal feature extraction becomes complex due to continuous changes that may occur during transitions between the wearer’s intended movements [18]. Consequently, domain-specific knowledge and trial-and-error approaches become necessary to derive meaningful features [9,19,20,21,22].

Deep learning (DL) technology has risen in popularity as a tool to autonomously detect users’ locomotion activities or intents in the field of human activity recognition (HAR) [18,23,24]. Unlike traditional machine learning (ML) techniques, DL significantly reduces the need for laborious extraction of valuable features from wearable sensor data. Particularly, convolutional neural networks (CNN), with their local dependency and scale invariance, have become the most widely used for many practical issues, such as image classification [25,26], object recognition [27], and natural language processing [28,29,30,31,32]. Several recent studies have formulated hybrid architectures by incorporating additional layers, such as long short-term memory (LSTM) [33,34,35,36], gated recurrent unit (GRU) [20,21,22,37], or squeeze-and-excitation network (SENet) [38]. These state-of-the-art technologies aim not only to minimize the computational cost (i.e., the number of parameters) but also to enhance prediction performance in HAR. While LSTM and GRU, variants of the recurrent neural network (RNN), can improve the accuracy of activity or intention recognition, they often entail issues such as prolonged training time. This is because the computational process of each subsequent stage depends on the result of the previous step and is executed sequentially. CNN has fewer parameters and quicker training than LSTM and GRU due to its local connectivity and weight-sharing mechanisms [22]. However, the capability and accuracy of feature extraction are contingent on the network’s depth. As the depth increases, the model parameters rise exponentially. Therefore, choosing the appropriate network depth in CNN or hybrid model architectures, such as CNN + LSTM (GRU) and LSTM + CNN, in addition to model hyperparameters, is critical.

In this paper, we introduce multivariate single and multi-head CNN architectures for human locomotion activity recognition while wearing a lower limb wearable robot. In our design, two CNN architectures with different network depths and convolutional filter sizes each maintain a fixed kernel size. These architectures extract local temporal features from multivariate signals acquired from EMGs and a wearable robot, respectively. Each architecture then connects to fully connected layers with varying neuron sizes and ultimately identifies five locomotor activities: level ground walking (LW), stair ascent (SA), stair descent (SD), ramp ascent (RA), and ramp descent (RD). These activities are measured across three terrains: flat ground, staircase, and ramp.

The main contributions of this study include: First, we collected prospective research data evaluating the locomotion activity of 500 healthy adults aged 19 to 64. Second, using different multivariate signals collected from eight electromyography (EMG) sensors and a wearable robot, we compared the prediction performance for five locomotor activities between our two CNN architectures and three competing models, namely a CNN and two hybrid architectures (i.e., CNN + LSTM and LSTM + CNN). Lastly, we demonstrated that by only using the encoder, i.e., hip angles and velocities and postural signals, i.e., roll/pitch/yaw from an inertial measurement unit (IMU) from the lower limb wearable robot, a deeper single-head CNN architecture significantly outperforms the three competing architectures.

The rest of this paper is organized as follows: Section 2 presents the related works. Section 3 explains data collection, the proposed CNN model architecture, and hyperparameter optimization. Section 4 describes the collected data characteristics and compares the proposed and three competing models. The conclusion and future study plans are summarized in Section 5.

## 2. Related Works

This section outlines several deep neural network (DNN) architectures used for detecting user locomotion activity and intent in wearable exoskeleton lower limb robots and HAR. Table 1 summarizes 10 of the most relevant studies that have attempted to develop a model for identifying human locomotion activity.

### 2.1. Locomotion Activity or Gesture Recognition

Ref. [39] proposed a deep CNN architecture, named ConvNet, to perform efficient and effective HAR using smartphone sensors. Their model leverages the inherent properties of activities and 1D time series signals, providing a way to automatically and adaptively extract robust features from raw data. Similar to our study, they adjusted structural hyperparameters using a greedy-wise tuning approach within the search space. This included the number of layers (1–4), the number of feature maps (10–200), and the filter size (1 × 3–1 × 15). They suggested the ConvNet configuration of C(96)–C(192)–C(192)–D(1000)–S(6) with a kernel/filter size of 9 and a pooling size of 3. Here, C represents the number of feature maps in convolutional/pooling layers, while D and S represent the number of nodes in the fully connected layers and the softmax layer, respectively. The ConvNet exhibited a superior recognition performance of 94.79% compared to other methods, using handcrafted features extracted from the UCI-HAR dataset [40]. Ref. [33] developed a new DNN framework, DeepConvLSTM, which combines four convolutional layers with two recurrent LSTM layers for identifying different activity modes on two public datasets, namely OPPORTUNITY [41] and Skoda [42]. They tested the performance of 12 different ML algorithms on the OPPORTUNITY dataset and two CNN models on the Skoda dataset. DeepConvLSTM outperformed the other methods in terms of F1 score on both datasets: achieving 89.5% and 91.5% in 5 locomotion modes and 18 gesture recognition for OPPORTUNITY, respectively; and 95.8% in gesture recognition for Skoda. To reduce model parameters and speed up convergence, Ref. [34] developed a DNN architecture (i.e., LSTM-CNN) with a global average-pooling (GAP) layer followed by a batch normalization layer (BN). In their proposed architecture, Ref. [34] examined the impact of several network structures (e.g., with/without the use of GAP and BN) and three hyperparameters (i.e., five optimizers, the number of filters, and batch size) using the UCI-HAR dataset. Their LSTM-CNN model, structured as L(32)–L(32)–C(64)–C(128)–GAP–BN, where L and C denote the number of nodes and feature maps in LSTM and convolutional layers, respectively, achieved the highest weighted F1 score compared to other models, such as CNN [43] and DeepConvLSTM [33]. The model achieved scores of 95.8% on the UCI-HAR, 92.71% on OPPORTUNITY, and 95.75% on WISDM datasets [44], respectively.

LSTM and GRU have shown similar performance in modeling speech signals and processing natural language. Generally, LSTM is more powerful and flexible than GRU with longer sequence data but is more complex and can tend to overfit. In contrast, GRU consumes less memory and has faster processing times compared to LSTM. Ref. [20] suggested a hybrid DNN classifier that combines two CNN and two stacked GRU layers to automatically extract spatial or local information from sensor data with different modalities, such as a gyroscope or accelerometer, gathered from Google Nexus or Samsung Galaxy S5 mobile devices and smartwatches. The hybrid CNN-GRU achieved classification accuracy between 90.44% and 96.54% on the WISDM smartphone, smartwatch activity, and biometrics dataset [45]. Similarly, some works [21,22,37] used 3-head CNN-GRU architectures to capture various temporal local dependencies in raw data for HAR, but different model structures. These structures varied in the order of combining layers (e.g., BN, dropout, max-pooling, or GAP), and the number and sizes of filters in the convolutional layers. The 3-head CNN-GRU models performed well on three datasets, yielding F1 scores between 96.19% and 96.71% for UCI-HAR, 96.39% and 97.22% for WISDM, and 95.24% and 96.59% for PAMAP2.

**Table 1:** Summary of related works on deep learning-based human locomotion activity detection.

Locomotion Activity or Gesture Recognition					
Model	Used Dataset (Sensors or Devices|Subjects)	Locomotion or/and Transition Mode	Hand-Crafted Features Use	Performance	Model Assessment
CNN [39]	UCI-HAR (3-axial linear acceleration and 3-axial angular velocity|30)	6 activity modes	Both	94.79 and 95.75 for without/with hand-crafted features, respectively	Accuracy
DeepConvLSTM [33]	OPPORTUNITY (7 IMUs and 12 3D acceleration|4); Skoda (10 3D acceleration|1)	5 locomotion and 18 gesture modes for OPPORTUNITY; 10 gesture modes for Skoda	No	OPPORTUNITY (89.5 and 91.5); Skoda (95.8)	Weighted F1 score (or measure)
LSTM-CNN [34]	UCI-HAR; OPPORTUNITY; WISDM (1 accelerometer|36)	6 activity modes for UCI-HAR; 18 gesture modes for OPPORTUNITY; 6 activity modes for WIDSM	No	UCI-HAR (95.8); OPPORTUNITY (92.71); WISDM (95.75)	Weighted F1 score
CNN-GRU [20]	WISDM smartphone and smartwatch activity and biometrics (3-axis accelerometer and 3-axis gyroscope|51)	18 activity modes	No	Smartwatch (96.54) and Smartphone (90.44)	Accuracy
Multi-input CNN-GRU [37]	UCI-HAR; WISDM; PAMAP2 (a heart rate monitor and 3 IMUs|9)	6 activity modes for UCI-HAR and WISDM respectively; 18 activity modes for PAMAP2	No	UCI-HAR (96.2 and 96.19); WISDM (97.21 and 97.22); PAMAP2 (95.27 and 95.24)	Accuracy and F1 score
Multichannel CNN-GRU [21]	UCI-HAR; WISDM; PAMAP2	6 activity modes for UCI-HAR and WISDM respectively; 18 activity modes for PAMAP2	No	UCI-HAR (96.67 and 96.72); WISDM (96.41 and 96.39); PAMAP2 (96.25 and 96.59)	Accuracy and F1 score
Multi-scale CNN-GRU [22]	UCI-HAR; WISDM; PAMAP2	6 activity modes for UCI-HAR and WISDM respectively; 18 activity modes for PAMAP2	No	UCI-HAR (96.71 and 96.72); WISDM (97.18 and 97.17); PAMAP2 (96.28 and 96.27)	Accuracy and F1 score
LDA [11]	14 EMGs, 4 goniometers, and 4 IMUs|10 able-bodied adults and one left traumatic above-knee amputee	5 locomotion activities and 8 transitional modes	Yes	1.43 for overall; 0.76 for steady-state; and 4.5 for transitional modes	Error rate
CNN [46]	4 IMUs and a load cell|7 healthy adults	5 locomotion activities and 8 transitional modes	No	97.64, with the average delay of 23.97% in the transitional modes	Accuracy
CNN with hierarchical classification layer [8]	7 IMUs|8 healthy adults	16 locomotion modes	No	94.34	Accuracy

### 2.2. Locomotion Intention Recognition

Ref. [11] showed that the inclusion of bilateral neuromechanical signals could significantly improve the accuracy of an intent recognition control system. The system could predict five locomotor activities (i.e., LW, RA, RD, SA, and SD) and eight transition modes, simply by adding one additional sensor from the contralateral side. Additionally, the authors demonstrated the feasibility of their approach by controlling the walk of a left traumatic above-knee amputee using a powered leg prosthesis in offline analysis. They achieved the lowest error rate (1.43, 0.76, and 4.5 in linear discriminant analysis (LDA) for overall, steady-state, and transitional modes, respectively) compared to other models, namely, support vector machines (SVM) and artificial neural networks (ANN). In a study focused on transitional movement intention recognition, ref. [46] collected movement activity data from seven healthy subjects wearing a soft lower limb exoskeleton robot fitted with four IMUs and a load cell. These data included five steady-state movements (i.e., LW, SA/SD, and RA/RD) and eight transitional modes (i.e., LW→SA, LW→SD, SA→LW, SD→LW, LW→RA, LW→RD, RA→LW, and RD→LW). They developed an IMU-based motion intention model called the DNN-based deep location mode identification model (DDLMI), comprising four convolutional layers followed by a fully connected layer. The model achieved a recognition rate of 97.64% for the five movements and an average delay of 23.97% for the eight transitional modes. In a separate study aimed at identifying user-initiated locomotion motions, ref. [8] designed a DNN classifier that combined stacked causal 2D convolutional layers followed by a fully connected layer. Unlike the above studies, this study emphasized the hierarchical classification of less specific locomotor activities before more specific actions to detect transitional motions. Using data from 16 locomotion modes collected from eight healthy adults, the authors demonstrated that their locomotion mode detection classifier was more effective, achieving an accuracy rate of 94.34%.

## 3. Methods

### 3.1. Participant Demographics and Recruitment Process

This study conducted a prospective analysis of five distinct locomotor activities—LW, SA, SD, RA, and RD—engaged in by 500 adults aged 19 to 64 years, from 1 September to 30 November 2022. We recruited participants through in-hospital advertisements targeting outpatients and their guardians. During recruitment, each participant was informed about the study’s objectives, the personal details to be collected (e.g., name, gender, residential area, date of birth, contact information), and the equipment and procedures for data collection. The exclusion criteria encompassed individuals who declined participation in the clinical study, those unable to walk independently, or those unable to communicate verbally.

### 3.2. Ethical Considerations

To address privacy and research ethics, we offered participants the following provisions: (1) Participants voluntarily agreed to join the clinical study without forfeiting any rights by signing the consent form. (2) While participant consent forms and other records might be accessed by research staff and pertinent agencies, all documents will remain confidential. (3) Participants consented to the use of portrait rights for photos and videos captured during physical data measurements as raw data for clinical research. Should consent be retracted, the associated data will be promptly deleted. (4) Participants have the liberty to rescind their consent for this clinical study at any point. All participants gave informed consent, encompassing the research subject consent form, security pledge, personal information collection and use agreement, and portrait rights use form. The study received approval from the Institutional Review Board (IRB) (No. GNUCH 2022-08-007-001) at Gyeongsang National University Hospital, Republic of Korea.

### 3.3. Data Collection

**Figure 1:**
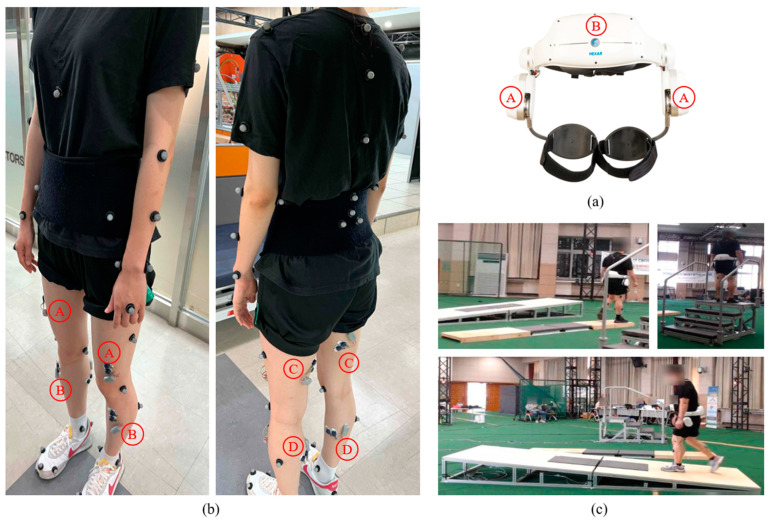
Data collection process. (**a**) Wearable lower-limb robot: Ⓐ–Left/Right Encoders and Ⓑ–IMU sensor. (**b**) EMG sensors and motion detection markers. The EMG sensor locations for detecting four lower limb muscles: Ⓐ–Vastus lateralis (VL), Ⓑ–Tibialis anterior (TA), Ⓒ–Biceps femoris (BF), and Ⓓ–Gastrocnemius lateralis (GAL). Markers present locations for measuring kinematic motion information, e.g., spine, shoulders, elbows, hands, feet, and ankles. (**c**) Gait courses with three terrains, namely, flat ground, stairs, and ramps. This figure shows an example of three locomotor activities: level ground walking (LW), ramp ascent (RA), and stair ascent (SA).

During the five locomotion behaviors, the participants, who were wearing a lower limb wearable robot, were instrumented with EMG sensors and a motion capture system in a simulated space for activities of daily living (ADL), as illustrated in Figure 1.

They performed the five locomotor activities on three types of terrains with the following specifications: (1) For the flat ground terrain, a total length of 3000 mm was set. (2) For the ramp terrain, a total length of 3600 mm, a total height of 400 mm, and a slope of 4.3 degrees were set. (3) For the staircase terrain, a total height of 692 mm was set with four steps and a full footrest depth of 1519 mm, which included each footrest depth of 303 mm for the first and third steps and a final footrest depth of 610 mm.

The Hector H30A wearable robot, produced by HEXAR Humancare, Republic of Korea, was employed in this study. The robot is designed to assist the hip joint’s muscle strength while walking on various terrains, such as flat, uphill, and downhill [47]. The robot comprises actuators, control units, sensors, and batteries and weighs approximately 4.3 kg. The two brushless DC (BLDC) motors in the robot are each capable of providing up to 12 Nm of torque to the user’s hip joint. The robot is equipped with two types of sensors: rotary encoders and an IMU. The encoders, placed within the actuator modules, measure the hip joint’s angular velocity. The IMU sensor, which includes a tri-axial accelerometer and a tri-axial gyroscope, is used to estimate the wearer’s posture. The robot can operate continuously for about 2 h. During the study, we collected 7-channel wireless signals at the lowest level (i.e., default mode) of the three torque modes that support the hip joint’s muscle strength. These signals, sampled at a rate of 71.42857 Hz, included the left/right hip angles (in degrees), left/right velocities (in rpm), and three postures (roll, pitch, and yaw; in degrees).

In addition to the robot’s sensor data, we used an 8-channel wireless surface electromyography (EMG) system (Delsys Trigno, Delsys, Inc., Boston, MA, USA), acquired at 2000 Hz [48], to acquire EMG signals from four lower limb muscles. These muscles included the vastus lateralis (VL), tibialis anterior (TA), biceps femoris (BF), and gastrocnemius lateralis (GAL) of both lower limbs [49]. Prior to placing the EMG sensors, the skin over each muscle was cleaned using alcohol wipes to remove dry skin and skin oils. The EMG electrodes were then affixed to the skin using double-sided adhesive tape, and their placement was adjusted as necessary. To measure kinematic motion information, an eight-camera motion capture system (Kestrel 2200, Motion Analysis Corp., Santa Rosa, CA, USA) was used. This system captured information about the spine, shoulders, elbows, hands, feet, and ankles, at a sampling rate of 100 Hz [50].

### 3.4. Model Architecture

**Figure 2:**
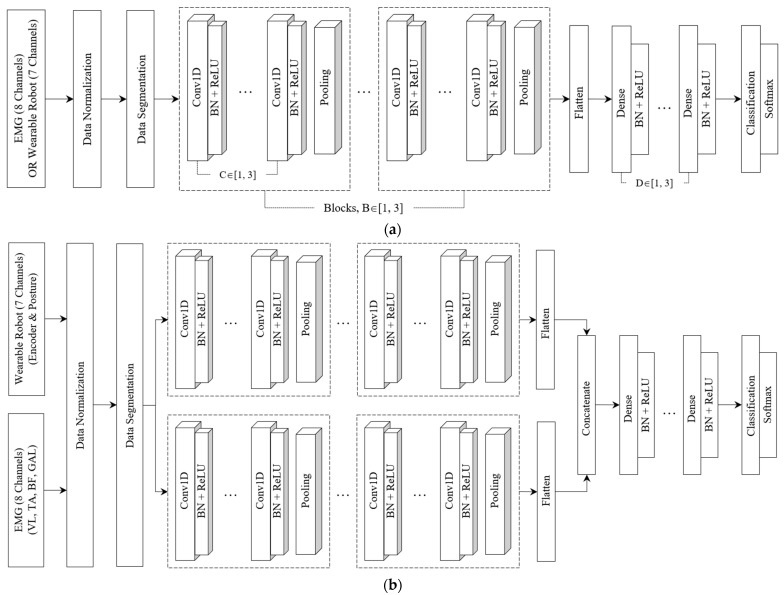
Single or multi-head CNN architecture for locomotor activity detection. Symbols B, C, and D denote the number of blocks, convolutions, and fully connected layers. (**a**) Single-head CNN architecture. (**b**) Multi-head CNN architecture.

The model architecture of the proposed model is described in Figure 2. It leverages either a single or multi-head CNN structure to extract richer features from the two types of multivariate signals gathered from the wearable robot and the EMG sensors. These architectures are similar in structure but vary in the number of blocks containing convolutional layers, filter sizes, and the number of fully connected layers.

In the single-head CNN architecture, each block—specifically, the feature extractor—captures local temporal features from EMG sensor signals and the wearable robot. Each can encompass up to three convolutional layers. We limited convolutional blocks to three to avoid degradation from potential gradient vanishing and exploding as network depth increases [51,52,53]. The number of filters in a convolutional layer varied among four sizes: 16, 32, 64, or 128, with adjacent convolutional layers having a twofold difference in feature maps. We employed a fixed kernel size of 3 with a stride of 1 to augment decision functions and ensure quicker network convergence with non-linear activations. To hasten training and convergence, a BN layer and a rectified linear unit (ReLU) activation followed each convolutional layer. Each block concluded with a pooling layer, facilitating down-sampling to minimize parameters, preserve dominant features, and filter noise from involuntary human body jitter [34]. We contemplated max-pooling or average-pooling layers with a pool size of two. Additionally, we restricted the number of fully connected layers to three. In the first fully connected layer, the number of neurons could be set to 32, 64, 128, 256, or 512. Similarly, adjacent layers exhibited a twofold difference in nodes, mirroring the design in the convolutional layer.

The multi-head CNN architecture, as displayed in Figure 2, was designed as a separable structure to independently preserve the unique characteristics of different signals from the EMG sensors or the wearable robot. The temporal features extracted from various blocks were combined to form the final feature representation. These features were then forwarded to the fully connected layers. A classifier with a softmax layer was then used to identify the five locomotor activities.

### 3.5. Hyperparameter Optimization

Hyperparameter optimization, also known as hyperparameter tuning, is the process of selecting the best combination of hyperparameters that maximizes the performance of a learning algorithm. Traditional methods such as grid search are exhaustive in their approach and involve trialing a subset of hyperparameter values to find the optimal configuration. However, due to the high number of trials required and the need to keep track of them, this approach can be quite time-consuming. More recently, alternative methods such as random search and Bayesian optimization have gained popularity. One specific Bayesian optimization method is the tree-structure parzen estimation (TPE) [54]. TPE is a unique Bayesian optimization method that sequentially builds models to estimate the performance of hyperparameters based on past measurements [55,56]. It utilizes conditional probability *P*(*x*|*y*), where *x* represents hyperparameters and *y* represents the quality score (e.g., loss, accuracy) on the objective function. This method offers the advantage of efficient convergence to a global minimum in a relatively shorter time.

In this study, our focus was on the structural optimization issues, more specifically, determining the depth of the convolutional and fully connected layers in the proposed architecture (i.e., the number of blocks, convolution, and fully connected layers). For this purpose, we employed the Hyperopt library [56,57] to identify hyperparameters that yield the highest identification ability in validation data. Subsequently, the predictive performance of our models, which are designed using these optimal hyperparameters, was evaluated on test data.

## 4. Results and Discussion

### 4.1. Experimental Setup

Before the experiment, participants underwent a gait test on three distinct terrains for approximately 10 min to familiarize themselves with the wearable robot. During this preparatory phase, coordinators monitored the signal quality from both the wearable robot and the EMG sensors. For data collection, participants were instructed to begin and conclude each of the five movement activities with their feet together, regardless of starting with the left or right foot. Each activity was performed thrice by every participant. Consequently, for each locomotor activity, we obtained nine data files per participant, encompassing details from the wearable robot, EMG sensors, and motion capture system [58]. Throughout the research, 78 cloud workers meticulously reviewed the motion-captured data, identifying specific gait events such as heel strikes and toe-offs. Additionally, they verified the commencement and conclusion timestamps for each locomotor activity based on the data from the EMG sensors and the wearable robot to ensure data integrity.

### 4.2. Data Characteristics

This study was conducted with a total of 500 participants, whose ages ranged between 19 and 64 years. The most represented age group was 30–49 years, with fewer participants in the 19–29 and 50–64 age groups. The average age was 40.16 ± 13.39 years, with a slight difference between males (40.02 ± 13.47) and females (40.29 ± 13.31). Gender distribution was evenly split with 250 males (50%) and 250 females (50%) (Table 2).

Table 3 shows the gait cycle periods for the different locomotor activities (LW, SA, SD, RA, and RD). The gait cycles exhibited regular periods: 1.39–1.4 s for LW; 1.57–1.58 s for SA; 1.51–1.53 s for SD; and 1.9–1.95 s for RA. However, the RD activity displayed slightly more variation, ranging from 1.62 to 1.7 s, with the toe-off events taking relatively longer than heel strikes.

Table 4 demonstrates the measurement time of the collected data from all participants who wore the wearable robot with EMG sensors and attempted five locomotor activities three times. The collected raw signals did not contain missing values. The sample size, as shown in Table 4, was (23,288,780, 8) for the EMG data and (832,447, 7) for the wearable robot data. The average measurement time for the collected multivariate signals was approximately 4.66 s. Among the locomotor activities, LW (approximately 4.8 s) and RA (approximately 4.9 s) took longer than the other activities, with SA and SD both taking approximately 4.6 s and RD taking the least time at 4.3 s.

### 4.3. Preprocessing

Raw data from sensors can be tainted by noises originating from various factors, including electronic fluctuations and sensor malfunctions. While signal processing techniques, such as the Butterworth filter [11,40], can be employed to eliminate these disturbances, caution is advised. Such techniques may inadvertently strip away crucial information from raw signals [59]. Furthermore, introducing new time series data into a pre-trained model demands extra efforts to address these artifacts, even if the latency is brief, using the same preprocessing steps. In our study, we utilized raw signals from EMG sensors and a wearable robot without applying any filtering. These raw signals were then normalized to a range between −1 and 1.

### 4.4. Data Segmentation

After normalization, the signals were passed to the segmentation phase, an essential step in preparing the data collected from sensors [19]. We segmented the signals into sequences using the overlapping sliding window technique [59], which is preferred for its straightforwardness and computational efficiency [19]. A window size of 1.76 s was employed, with an overlap ratio of 0.9, determined by the average value and standard deviation of the left heel strike (LHS). This windowing method was applied to the multivariate signals sourced from both the EMGs and the wearable robot. This choice was made considering the different measurement times recorded during five locomotor activities, as shown in Table 4. Typically, the sequences generated after sliding window segmentation are randomly divided into training and test sets. However, this conventional data partitioning approach can lead to sequences from the same user’s activity appearing in both the training and test sets, causing information leakage. To prevent this, we applied a group-based data partitioning strategy [21,22,33,39] to ensure that samples from the same user only exist in one of the datasets. This approach divided the sequences into training, validation, and test sets with a sample ratio of 8:1:1. The distribution of sequence data, including the number of users, sample sizes, and locomotor activity frequencies in the training, validation, and test sets used in the experiment, is provided in Table 5.

### 4.5. Benchmark Models

We considered three well-known models: the CNN-based model, called DDLMI [46]; the CNN-LSTM model; called DeepConvLSTM [33]; and the LSTM-CNN model [34], which are applied to the application domains of motion intention detection or HAR. The DDLMI architecture integrates four convolutional layers activated by ReLU, complemented by four max-pooling layers and a subsequent fully connected layer, which is then succeeded by both a BN and a dropout layer. We utilized the softmax function to determine the model’s probability. DeepConvLSTM, on the other hand, encompasses four sequential convolutional layers and a pair of LSTM layers, culminating in a softmax layer. Within each convolutional segment, ReLU was harnessed to delineate the feature maps. Notably, the recurrent units’ activation was discerned using the hyperbolic tangent function. The LSTM-CNN model blends two LSTM layers and a duo of convolutional layers, bridged by a max-pooling layer. Post the final convolutional layer, a GAP is situated, succeeded by a BN layer. The model concludes its processing by yielding an output from a fully connected layer, equipped with a softmax classifier. Experiments were performed using a segmented dataset in Table 5 under identical conditions using the model structure and learning parameters provided in the above studies.

### 4.6. Experimental Environments and Implementation

We used the Keras API of the Tensorflow backend to implement the proposed model and benchmark models. The experiments were carried out on a system with an Intel Xeon(R) Silver 4208 @ 2.1 GHz CPU, NVIDIA Quadro RTX A6000, running Ubuntu 22.04 LTS. The code was written in Python 3.8.16, using the Intellij IDEA 2019.2.4 (Ultimate Edition), and leveraging Tensorflow-GPU 2.5, NumPy 1.19.5, Pandas 1.4.4, Matplotlib 3.5.3, and Hyperopt 0.2.7.

### 4.7. Evaluation Metrics

The model performance was evaluated using four statistical criteria [21,22]: accuracy, recall, precision, and F-measure. These metrics can be mathematically defined as follows:

(1) $$\mathrm{Accuracy}=\frac{TP+TN}{TP+FP+FN+TN}$$

(2) $$\mathrm{Recall}=\frac{TP}{TP+FN}$$

(3) $$\mathrm{Precision}=\frac{TP}{TP+FP}$$

(4) $$\mathrm{F-}\mathrm{measure}=2\times \frac{\mathrm{Precision}\times \mathrm{Recall}}{\mathrm{Precision}+\mathrm{Recall}}$$

In Equations (1)–(4), *TP*, *FP*, *FN*, and *TN* represent true positive, false positive, false negative, and true negative values, respectively. In addition to these metrics, a confusion matrix was also used to illustrate the classification results for each locomotor activity.

### 4.8. Experiments on Different Network Architectures

We performed three distinct experiments on the dataset to investigate the benefits of utilizing both different multivariate signals collected from EMG sensors and a wearable robot: First, the locomotor activity recognition performance of the proposed model was evaluated using only the multivariate signals gathered from EMG sensors. The results were then compared with the performance of the three benchmark models. Second, the locomotor activity recognition performance of the proposed model was evaluated using only the multivariate signals from the wearable robot. Again, the results were compared with the benchmark models. Lastly, the performance of the proposed model was examined when both types of multivariate signals (from the EMG sensors and the wearable robot) were considered together. In all three experiments, the hyperparameters of the proposed architecture (see Figure 2) such as the number of blocks and convolutional layers in each block, were optimized in the search space as indicated in Table 6, using the Hyperopt Python library [57]. During the hyperparameter optimization process, we set a limit on the number of candidate models (or evaluators) to 50. The model with the highest F-measure on the validation data was selected as the best model.

#### 4.8.1. EMG-Based Locomotor Activity Detection

**Figure 3:**
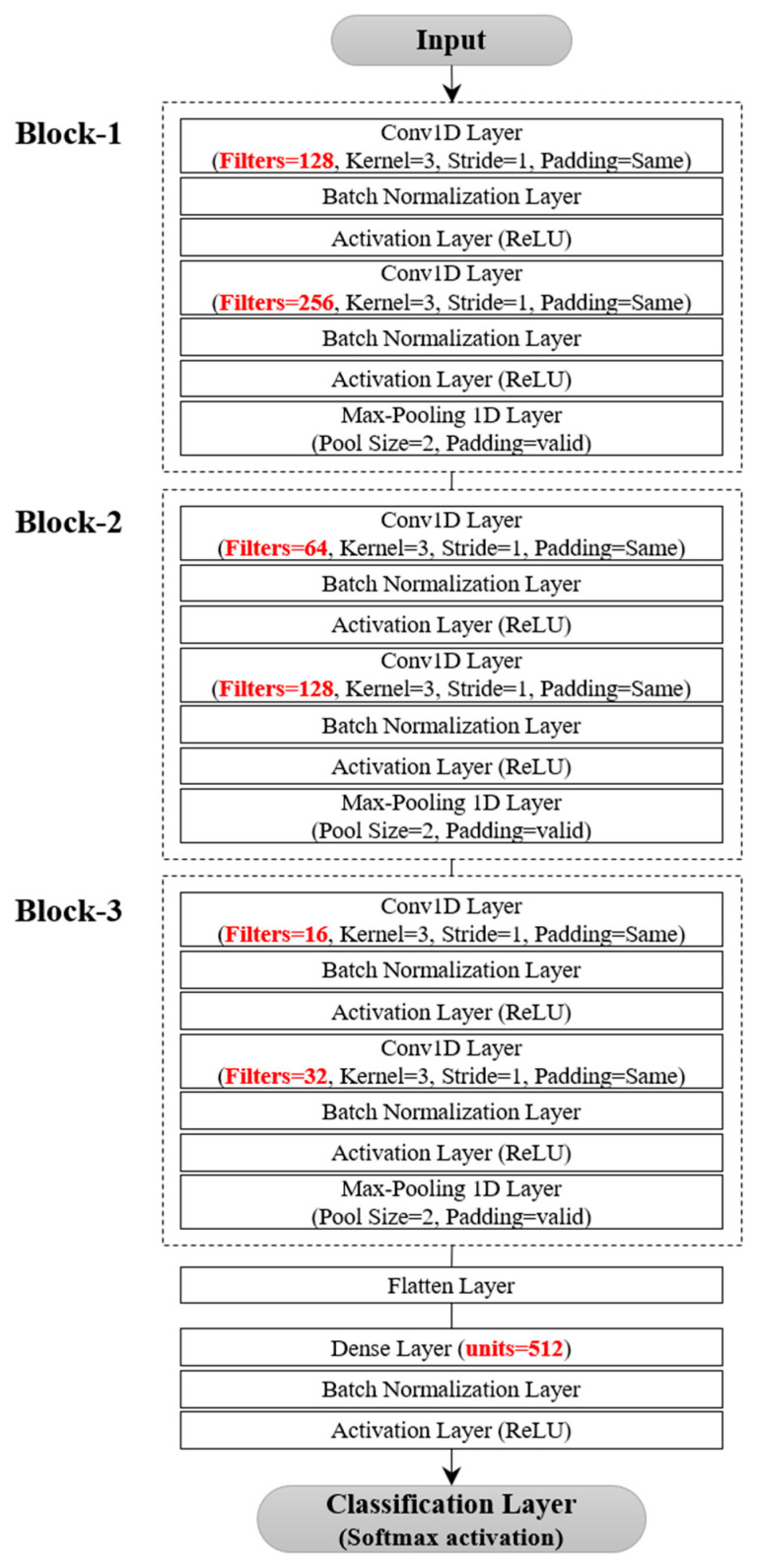
Single-head CNN architecture for EMG dataset.

Following the hyperparameter optimization process, a single-head CNN architecture was determined to be the most effective model for detecting five locomotor activities from EMG data, as shown in Figure 3.

The architecture included three blocks and a fully connected layer. Each block consisted of two convolutional layers followed by a max-pooling layer, and the fully connected layer contained 512 units. The learning rate and batch size were set at 1 × 10^−4^ and 128, respectively. A detailed performance comparison was made between the proposed and the three benchmarking models by observing the variations in accuracy and loss during training and validation. The learning rate was reduced by a factor of 0.9 each time the validation loss of the model did not improve for 10 consecutive epochs in 200 epochs. The initial learning rate was set to 1 × 10^−4^ for DDLMI and the proposed model and 1 × 10^−3^ for DeepConvLSTM and LSTM-CNN model. The weights of each network were recorded when the model achieved its lowest validation loss.

**Figure 4:**
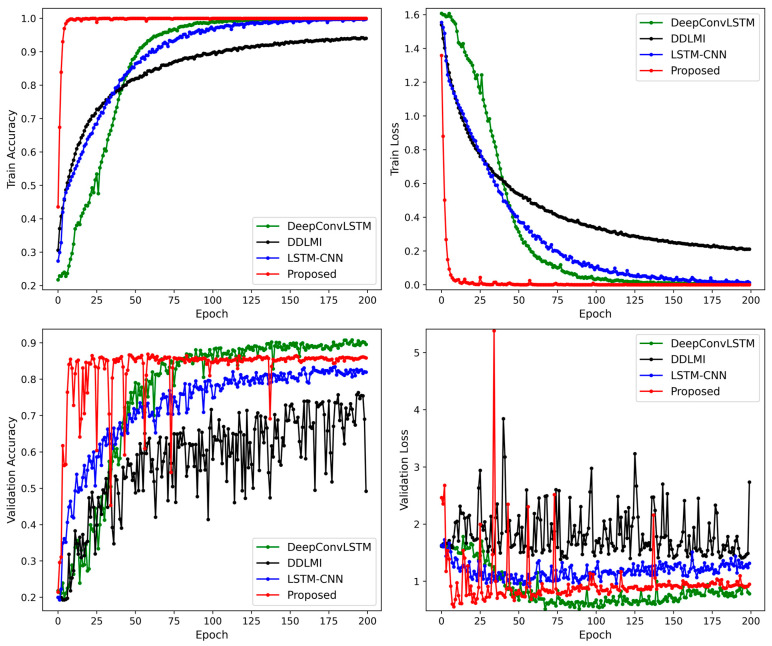
Train and validation accuracy plot for four models on EMG dataset.

Figure 4 demonstrates the alterations in accuracy and losses in the training and validation sets for the four models. The proposed model’s validation loss dropped rapidly in less than 20 epochs. In contrast, the two hybrid models, DeepConvLSTM and LSTM-CNN, displayed convergence trends after 50–75 iterations. DDLMI, however, did not converge. The epochs with the lowest validation losses were recorded as follows: 162-th epoch (1.373) for DDLMI, 67-th epoch (0.5136) for DeepConvLSTM, 57-th epoch (0.909) for LSTM-CNN, and 8-th epoch (0.5531) for the proposed model.

In the performance comparison provided in Table 7, our model can be seen exhibiting an accuracy of 0.8938, recall of 0.8943 (0.0779), precision of 0.8968 (0.0394), and F-measure of 0.8931 (0.0321) when applied to the EMG test data. Our method demonstrated superior performance over the other two models in extracting discriminative features and achieving accurate recognition results, even though the F-measure was slightly lower (by 0.49%) than DeepConvLSTM. When comparing processing times, DDLMI consistently stood out, boasting the swiftest average speed for both training epoch and inference time on test data. In terms of the training epoch, our model zipped ahead, being approximately 4.6 and 2.4 times faster than DeepConvLSTM and LSTM-CNN, respectively. Moreover, when it came to inferring test data, our proposed model demonstrated an impressive response time, roughly 3.7 and 2.6 times quicker than that of DeepConvLSTM and LSTM-CNN, respectively.

**Figure 5:**
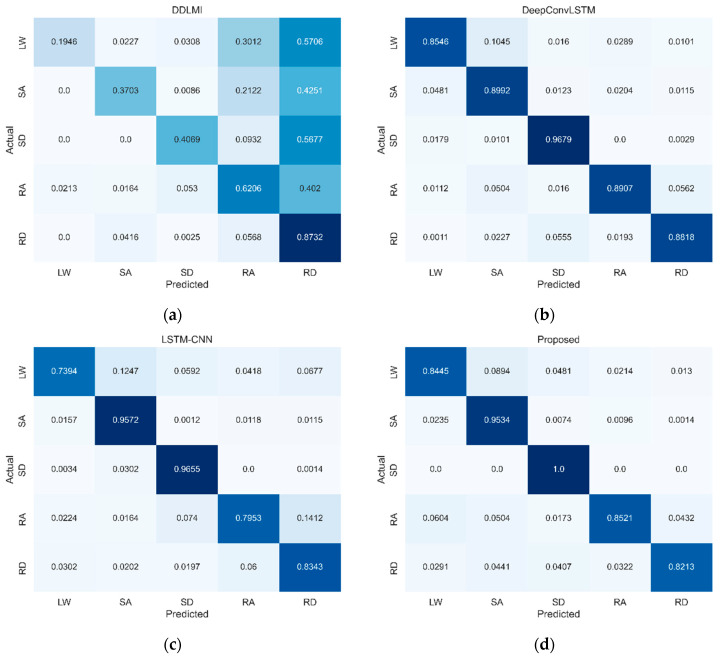
Confusion matrices for four models on EMG test dataset. LW, level ground walking; SA, stair ascent; SD, stair descent; RA, ramp ascent; RD, ramp descent. (**a**) DDLMI; (**b**) DeepConvLSTM; (**c**) LSTM-CNN; (**d**) Proposed. Darker blue indicates higher classification performance.

The confusion matrix (Figure 5) reveals that three activities, LW, RA, and RD, had higher misclassification rates in the DeepConvLSTM, LSTM-CNN, and our model. Specifically, the LW activity was often misclassified as SA: 10% for DeepConvLSTM, 12% for LSTM-CNN, and 9% for our model. For SA activity, 5% for DeepConvLSTM, 2% for LSTM-CNN, and 2% for our model were predicted to be LW activity. This outcome suggests that LW is similar to SA, causing some confusion in classification.

#### 4.8.2. Wearable Robot-Based Locomotor Activity Detection

**Figure 6:**
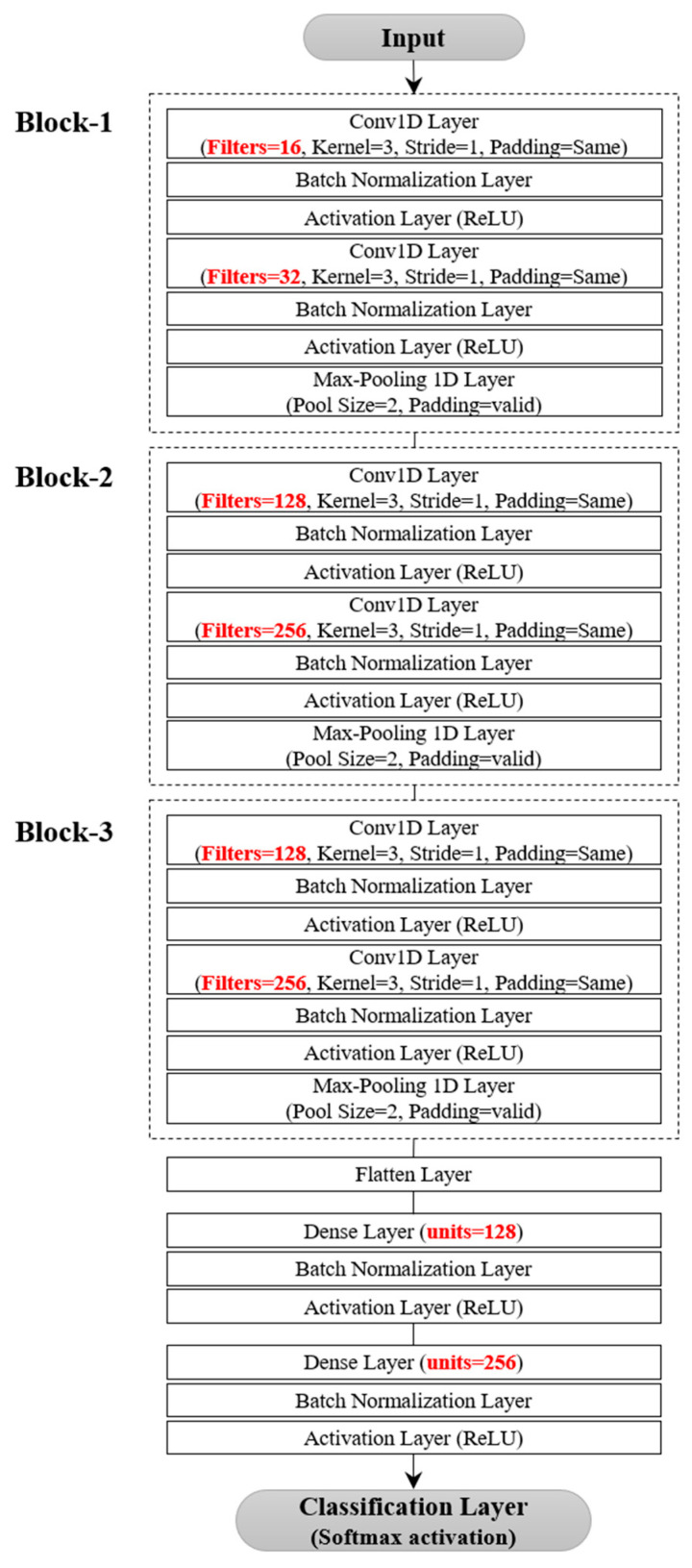
Single-head CNN architecture for wearable robot dataset.

**Figure 7:**
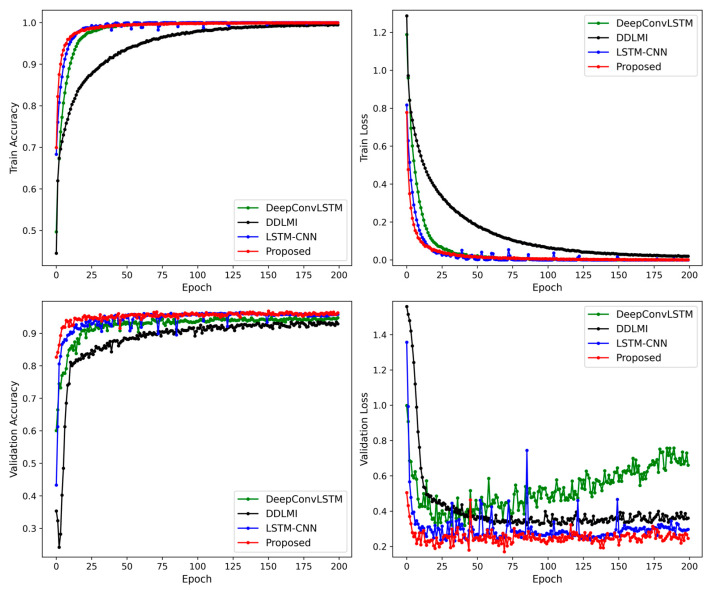
Train and validation accuracy plot for four models on wearable robot dataset.

Figure 6 presents a single-head CNN architecture designed to detect five locomotor activities from multivariate signals collected by a wearable robot. The model’s structure was similar to the one used for EMG data detection, but it had two fully connected layers with 128 and 256 units. The learning rate and the batch size were chosen to be 1 × 10^−4^ and 32, respectively. The training and validation dataset’s accuracy and loss changes were monitored for our model and the other three models, as depicted in Figure 7.

Compared to the previous results depicted in Figure 4, all four models exhibited more stable learning. In particular, the proposed model and LSTM-CNN converged faster. However, after 20–50 training epochs, DeepConvLSTM saw an increase in validation losses. The lowest validation loss was as follows: 102-th epoch (0.3203) for DDLMI, 22-th epoch (0.3061) for DeepConvLSTM, 34-th epoch (0.2321) for LSTM-CNN, and 69-th epoch (0.1706) for the proposed model.

The results of the performance comparison, are shown in Table 8, our model achieved the highest F-measure of 0.9617, surpassing other competing models: 0.9068 for DDLMI, 0.9441 for DeepConvLSTM, and 0.9557 for LSTM-CNN. Moreover, our model demonstrated accuracy improvements of 5.33%, 1.72%, and 0.63% over DDLMI, DeepConvLSTM, and LSTM-CNN, respectively. In alignment with the earlier processing time findings, DDLMI emerged as the quickest, trailed by our proposed model, and then by the two hybrid models, LSTM-CNN and DeepConvLSTM. The proposed model, LSTM-CNN, and DeepConvLSTM respectively clocked in average training epoch times of 19.21 s, 31.17 s, and 46.16 s, and inference times of 1.14 s, 2.51 s, and 2.89 s. Consequently, our model demonstrated a brisker inference speed, approximately 2.2 and 2.5 times faster than LSTM-CNN and DeepConvLSTM, respectively.

**Figure 8:**
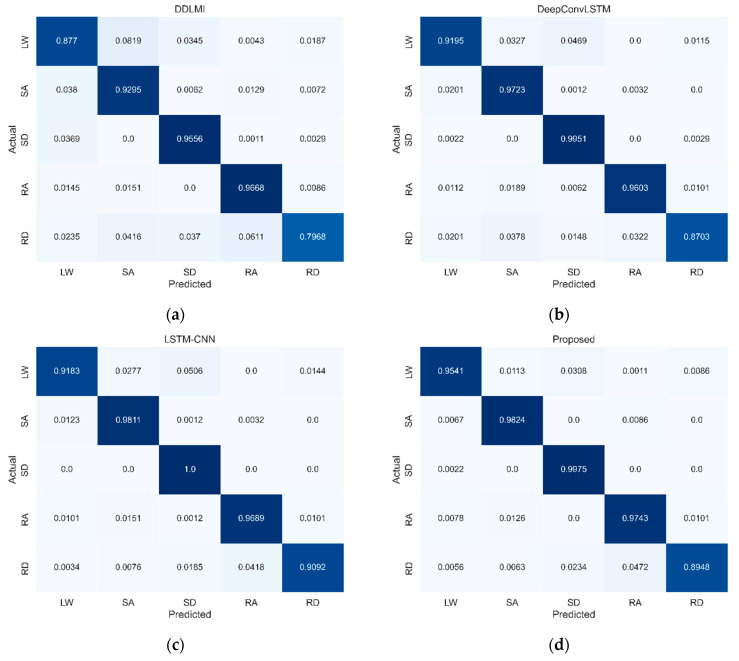
Confusion matrices for four models on wearable robot test dataset. LW, level ground walking; SA, stair ascent; SD, stair descent; RA, ramp ascent; RD, ramp descent. (**a**) DDLMI; (**b**) DeepConvLSTM; (**c**) LSTM-CNN; (**d**) Proposed. Darker blue indicates higher classification performance.

Figure 8 presents the confusion matrices’ differences when identifying the five locomotor activities using multivariate signals collected from the wearable robot. All four models had the lowest recognition performance for two activities: LW and RD. In the LW activity, three models showed high misclassification rates as the SD: 5% for DeepConvLSTM, 5% for LSTM-CNN, and 3% for our model, respectively. However, only for DDLMI, the misclassification rate was 8% in the SA activity. Moreover, the RD activity was misclassified as two activities, SA and RA: 4% and 6% for DDLMI and 4% and 3% for DeepConvLSTM. Meanwhile, the other two models, LSTM-CNN and our model, showed misclassification rates of 4% and 5% in the RA activity, respectively.

#### 4.8.3. EMGs and Wearable Robot-Based Locomotor Activity Detection

**Figure 9:**
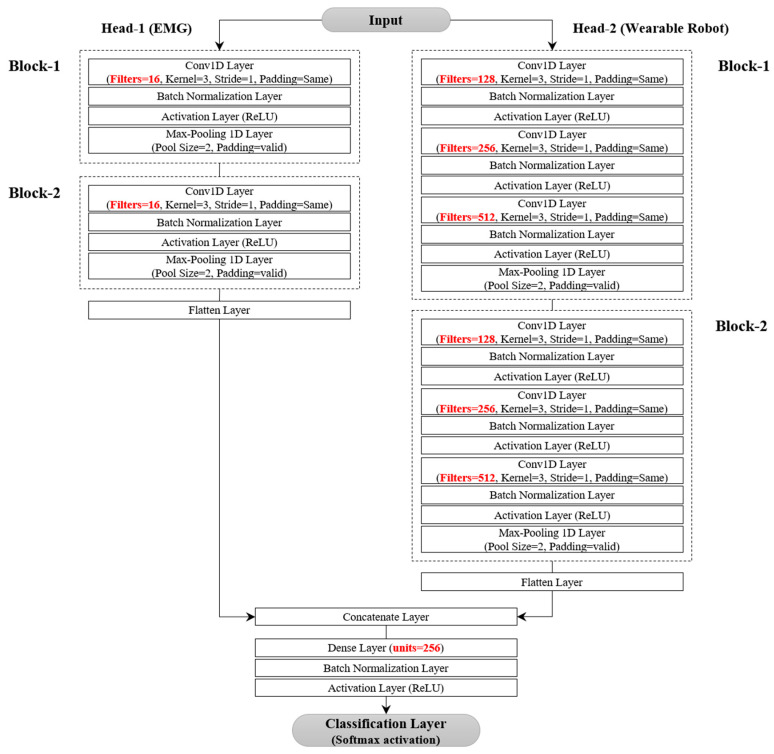
Multi-head CNN architecture for EMG and wearable robot datasets.

Figure 9 displays a two-head CNN architecture generated after hyperparameter optimization to identify the five locomotor activities. These activities were identified using multivariate signals collected from both EMG sensors and a wearable robot. In Figure 9, the first head consists of two blocks, each containing a convolutional layer followed by a max-pooling layer. The second head features a stacked structure, each comprising three convolutional layers with different filters, followed by a max-pooling layer. Both heads are connected to a fully connected layer via a concatenation layer. The learning rate and batch size were selected as 1 × 10^−4^ and 128, respectively.

**Figure 10:**
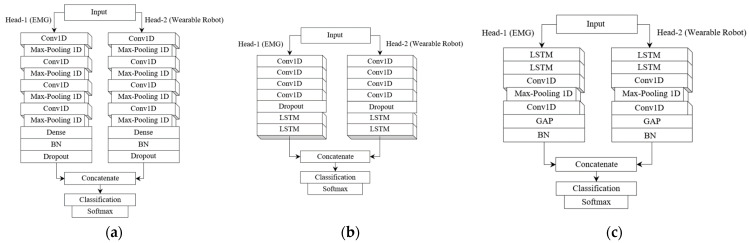
Two-head input architectures for DDLMI, DeepConvLSTM, and LSTM-CNN. (**a**) A modified version of DDLMI; (**b**) A modified version of DeepConvLSTM; (**c**) A modified version of LSTM-CNN.

To differentiate the multivariate signals of the two types, we modified the structure of the three competing models into a dual-head input architecture, as demonstrated in Figure 10.

**Figure 11:**
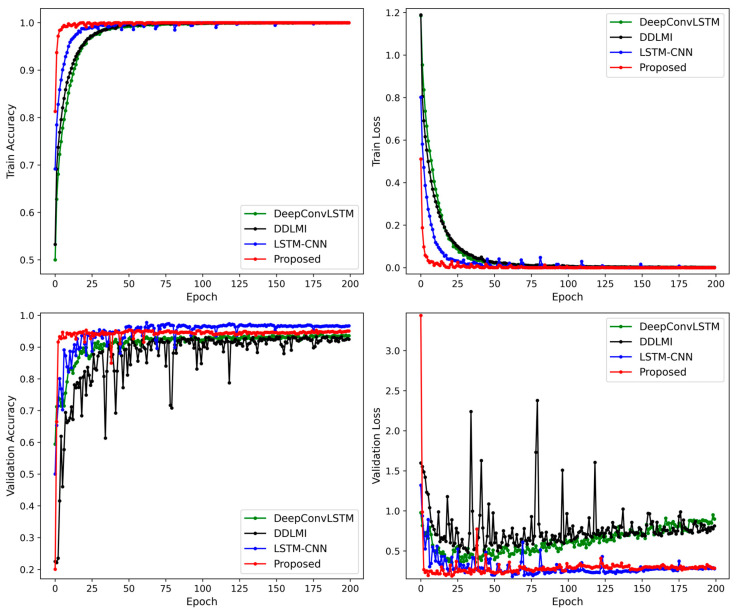
Train and validation accuracy plot for four models on EMG and wearable robot datasets.

We maintained their original structures as closely as possible. Figure 11 shows the changes in accuracy and losses in our model’s training and validation datasets, alongside the three competing models. From the experimental results, our model and LSTM-CNN showed more stable loss curves in the validation dataset compared to the other two models, DeepConvLSTM and DDLMI. The best validation loss was recorded as follows: 32-th epoch (0.4839) for DDLMI, 21-th epoch (0.3602) for DeepConvLSTM, 62-th epoch (0.186) for LSTM-CNN, and 21-th epoch (0.1908) for the proposed model.

As shown in Table 9, our model achieved an F-measure performance of 0.9539, representing a decrease of 0.72% from LSTM-CNN but increases of 3.74% and 2.25% from DDLMI and DeepConvLSTM, respectively. The proposed model demonstrated the fastest average training time (10.9 s) and inference speed (1.67 s) in the processing time comparison. Nonetheless, the trio of models exhibited a relatively slower learning pace and inference duration compared to the previous findings (refer to Table 7 and Table 8) due to the presence of a dual-head input structure. Particularly, the inference duration of DeepConvLSTM proved to be approximately 1.1 times slower (i.e., 8.11 s → 9.16 s) than that of the EMG sensors and 3.16 times slower (2.89 s → 9.16 s) than that of the wearable robot.

**Figure 12:**
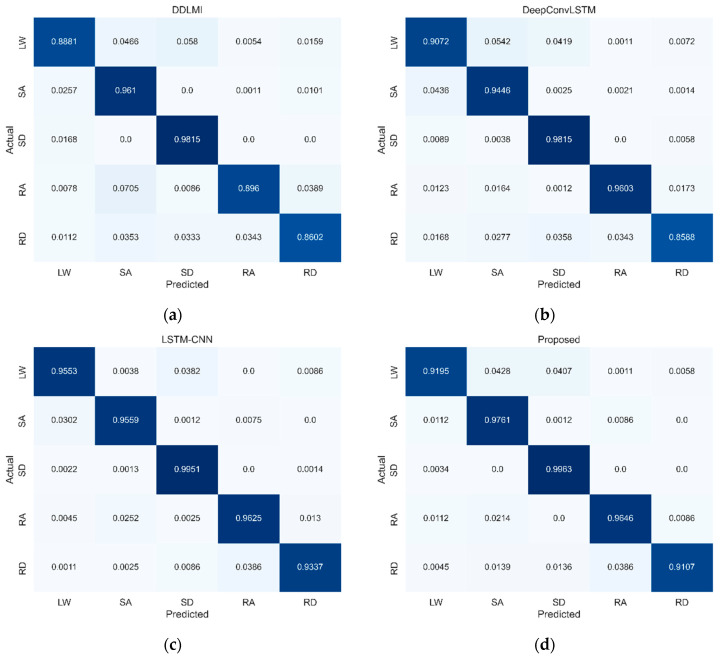
Confusion matrices for four models on EMG and wearable robot test datasets. LW, level ground walking; SA, stair ascent; SD, stair descent; RA, ramp ascent; RD, ramp descent. (**a**) DDLMI; (**b**) DeepConvLSTM; (**c**) LSTM-CNN; (**d**) Proposed. Darker blue indicates higher classification performance.

Figure 12 highlights the differences in the confusion matrices for identifying the five types of locomotor activity across the four models. All four models had the lowest detection performance for the RD activity. DDLMI and DeepConvLSTM had similarly high misclassification rates in three locomotor activities, SA, SD, and RA: 4%, 3%, and 3%, respectively, for DDLMI and 3%, 4%, and 3%, respectively, for DeepConvLSTM. Furthermore, our model and LSTM-CNN demonstrated the highest misclassification rate of 4% in RA.

#### 4.8.4. Summary

This study explored the predictive performance of five different locomotor activities across four distinct DNN architectures. The models were trained on different multivariate signals sourced from both EMG sensors and a wearable robot. All four models were more effective in identifying the five locomotor activities using encoder and posture signals (i.e., hip angles, velocities, roll/pitch/yaw) from the wearable robot, rather than the EMG sensors. F-measure performance improvements were as follows: 0.4742 to 0.9068 for DDLMI, 0.898 to 0.9441 for DeepConvLSTM, 0.8527 to 0.9557 for LSTM-CNN, and 0.8931 to 0.9617 for the proposed model (see Table 7 and Table 8). These results align with previous results, that highlighted high recognition accuracy in detecting human locomotor modes with IMU sensors [8]. Interestingly, when incorporating all signals from both the EMG sensors and the wearable robot, DDLMI and LSTM-CNN showed slight improvements in F-measure performance (0.97% and 0.54% increase, respectively). However, the proposed model and DeepConvLSTM displayed a slight decrease in performance (0.78% and 1.27%, respectively; see Table 8 and Table 9). The proposed model provided the highest predictive performance with an inference speed of 1.14 s (i.e., average 0.28 ms) in correctly identifying the five locomotor activities, achieving 0.9627 and 0.9617 in accuracy and F-measure, respectively. This model is cost-effective as it uses a smaller number of those multivariate signals compared to LSTM-CNN, as shown in Table 10.

## 5. Conclusions

In this paper, we proposed a multivariate single and multi-head CNN architecture to detect a user’s locomotor activity while wearing a lower limb wearable robot. Our research involved 500 healthy adult participants in an ADL space between 1 September and 30 November 2022. The prospective data were collected for the identification of five locomotor activities (LD, SA, SD, RA, and RD) across three terrains: flat ground, staircase, and ramp. Through our experiments, we compared the prediction performance between our proposed CNN and three other competing models. These models were trained on multivariate signals of different modalities, acquired from EMGs and a wearable robot. We found that a deeper CNN architecture outperformed the three competing models when using only the wearable lower limb robot’s encoder (hip angles and velocities) and postural signals (roll/pitch/yaw from an IMU). Despite the promising results achieved by the proposed CNN architecture, there remains room for improvement. Notably, our CNN model possesses a deep structure, which results in a higher computational cost. In subsequent research, we intend to employ lightweight models that integrate a GAP layer with either SENet or attention networks. This will enhance the efficiency of locomotion intent recognition across various continuous terrain scenarios and foster adaptive control profile generation for muscle strength support using the wearable lower limb robot.

## Figures and Tables

**Table 2:** Demographic characteristics.

Variable	Age Distribution			Total
	19–29	30–49	50–64	
Total, N (%)	150 (30)	200 (40)	150 (30)	500
Age (years), mean (SD)	24.49 (2.54)	39.28 (5.77)	57.01 (4.08)	40.16 (13.39)
Male	24.28 (2.25)	39.13 (6.03)	56.97 (3.98)	40.02 (13.47)
Female	24.69 (2.79)	39.42 (5.52)	57.05 (4.21)	40.29 (13.31)
Gender, N (%)				
Male	75 (50)	100 (50)	75 (50)	250 (50)
Female	75 (50)	100 (50)	75 (50)	250 (50)

SD, standard deviation.

**Table 3:** Gait cycle periods for five locomotor activities (units, s).

Gait Phase Patterns	Level Ground Walking	Stair Ascent	Stair Descent	Ramp Ascent	Ramp Descent	Total
LHS ^†^, mean (SD)	1.4 (0.33)	1.57 (0.4)	1.51 (0.32)	1.93 (0.47)	1.62 (0.48)	1.58 (0.17)
RHS ^‡^, mean (SD)	1.4 (0.32)	1.58 (0.41)	1.52 (0.3)	1.95 (0.47)	1.63 (0.51)	1.61 (0.18)
LTO ^§^, mean (SD)	1.39 (0.29)	1.57 (0.38)	1.53 (0.3)	1.95 (0.46)	1.69 (0.5)	1.61 (0.18)
RTO ^¶^, mean (SD)	1.4 (0.3)	1.57 (0.39)	1.51 (0.31)	1.9 (0.45)	1.7 (0.51)	1.61 (0.18)

^†^ LHS, left heel strike; ^‡^ RHS, right heel strike; ^§^ LTO, left toe-off; ^¶^ RTO, right toe-off. SD, standard deviation.

**Table 4:** Measurement time for five locomotor activities (units, s).

Device	Sample Size	Level Ground Walking	Stair Ascent	Stair Descent	Ramp Ascent	Ramp Descent	Total
EMG, mean (SD)	(23,288,780, 8)	4.81 (1.52)	4.65 (1.12)	4.6 (1.1)	4.9 (1.36)	4.34 (1.3)	4.66 (1.3)
Wearable robot, mean (SD)	(832,447, 7)	4.8 (1.51)	4.64 (1.11)	4.62 (1.11)	4.91 (1.37)	4.33 (1.32)	4.66 (1.31)

SD, standard deviation. The values enclosed in parentheses typically denote the number of samples and channels.

**Table 5:** Sequence data distribution in training, validation, and test sets.

Dataset	Users	EMG	Wearable Robot	Locomotion Activity Distribution
Training	400	(32,951, 3520, 8)	(32,951, 125, 7)	Level ground walking (6873), Stair ascent (6576), Stair descent (6387), Ramp ascent (7153), Ramp descent (5962)
Validation	50	(4120, 3520, 8)	(4120, 125, 7)	Level ground walking (862), Stair ascent (860), Stair descent (883), Ramp ascent (823), Ramp descent (692)
Test	50	(4126, 3520, 8)	(4126, 125, 7)	Level ground walking (894), Stair ascent (794), Stair descent (811), Ramp ascent (933), Ramp descent (694)

In the EMG and wearable robot datasets, the values enclosed in parentheses represent the number of samples, timestamps, and channels. Furthermore, in the context of activity distribution, the indicated value corresponds to the frequency of the respective locomotor activity.

**Table 6:** Search space of hyperparameters.

Hyperparameters			Selected Values or Ranges
Architecture	Block	Number of blocks	[1, 3]
	Convolution	Number of layers	[1, 3]
		Filters	16, 32, 64, 128
		Kernel size	3
		Stride	1
		Padding	Same
	Pooling	Type	Max-pooling, Average-pooling
		Pooling size	2
		Padding	Valid
	Dense	Number of layers	[1, 3]
		Number of neurons	32, 64, 128, 256, 512
	Batch normalization		Yes
Training	Optimizer		Adam
	Learning rate		1 × 10^−4^, 1 × 10^−3^
	Batch size		32, 64, 128, 256
	Epochs		20

**Table 7:** Performance comparison of the proposed model with three benchmark models on EMG dataset.

Model	Test Performance				Parameters			Processing Time (s)	
	Accuracy	Recall	Precision	F-Measure	Trainable	Non-Trainable	Total	Avg. Time per Training Epoch	Inference Time
DDLMI	0.4806	0.4931 (0.2609)	0.6649 (0.2561)	0.4742 (0.0933)	1,343,867	576	1,344,443	11.29	1.61
DeepConvLSTM	0.8982	0.8989 (0.0421)	0.8989 (0.0408)	0.898 (0.0278)	295,301	0	295,301	134.11	8.11
LSTM-CNN	0.8543	0.8583 (0.0999)	0.8545 (0.0458)	0.8527 (0.0454)	49,477	256	49,733	69.65	5.68
Proposed	0.8938	0.8943 (0.0779)	0.8968 (0.0394)	0.8931 (0.0321)	7,397,717	2272	7,399,989	29.17	2.21

**Table 8:** Performance comparison of the proposed model with three benchmark models on wearable robot dataset.

Model	Test Performance				Parameters			Processing Time (s)	
	Accuracy	Recall	Precision	F-Measure	Trainable	Non-Trainable	Total	Avg. Time per Training Epoch	Inference Time
DDLMI	0.9094	0.9051 (0.0689)	0.9121 (0.0339)	0.9068 (0.0343)	121,253	576	121,829	11.61	0.85
DeepConvLSTM	0.9455	0.9435 (0.0493)	0.9466 (0.0228)	0.9441 (0.0196)	294,981	0	294,981	46.16	2.89
LSTM-CNN	0.9564	0.9555 (0.0398)	0.9573 (0.0168)	0.9557 (0.0122)	49,349	256	49,605	31.17	2.51
Proposed	0.9627	0.9606 (0.04)	0.964 (0.0163)	0.9617 (0.0162)	838,245	2400	840,645	19.21	1.14

**Table 9:** Performance comparison of the proposed model with three benchmark models on EMG and wearable robot datasets.

Model	Test Performance				Parameters			Processing Time (s)	
	Accuracy	Recall	Precision	F-Measure	Trainable	Non-Trainable	Total	Avg. Time per Training Epoch	Inference Time
DDLMI	0.9176	0.9174 (0.0515)	0.9185 (0.0355)	0.9165 (0.0186)	1,465,115	1152	1,466,267	12.01	1.72
DeepConvLSTM	0.9329	0.9305 (0.0484)	0.934 (0.0279)	0.9314 (0.0238)	590,277	0	590,277	144.28	9.16
LSTM-CNN	0.9612	0.9605 (0.0222)	0.9621 (0.0098)	0.9611 (0.0071)	98,821	512	99,333	78.69	6.27
Proposed	0.9542	0.9534 (0.0369)	0.9556 (0.0221)	0.9539 (0.0113)	8,858,725	4160	8,862,885	10.9	1.67

**Table 10:** Best performance comparison of the proposed model with three benchmark models.

Model	Selected Inputs	Accuracy	Recall	Precision	F-Measure
DDLMI	EMG, Wearable robot	0.9176	0.9174 (0.0515)	0.9185 (0.0355)	0.9165 (0.0186)
DeepConvLSTM	Wearable robot	0.9455	0.9435 (0.0493)	0.9466 (0.0228)	0.9441 (0.0196)
LSTM-CNN	EMG, Wearable robot	0.9612	0.9605 (0.0222)	0.9621 (0.0098)	0.9611 (0.0071)
Proposed	Wearable robot	0.9627	0.9606 (0.04)	0.964 (0.0163)	0.9617 (0.0162)

## Data Availability

The raw data that underpin this study were published on the AI integration platform, AI Hub (https://aihub.or.kr/aihubdata/data/view.do?currMenu=115&topMenu=100&aihubDataSe=realm&dataSetSn=71526 (accessed on 8 August 2023)), which is operated by the National Information Society Agency (NIA) under the auspices of the Ministry of Science and ICT (MSIT). For further details about the use of these data, please contact the data management representative at AI Hub.

## References

[1] Mooney L.M., Rouse E.J., Herr H.M. (2014). Autonomous exoskeleton reduces metabolic cost of human walking during load carriage. J. Neuroeng. Rehabil..

[2] Zhang J., Fiers P., Witte K.A., Jackson R.W., Poggensee K.L., Atkeson C.G., Collins S.H. (2017). Human-in-the-loop optimization of exoskeleton assistance during walking. Science.

[3] Chen G., Qi P., Guo Z., Yu H. (2016). Mechanical design and evaluation of a compact portable knee–ankle–foot robot for gait rehabilitation. Mech. Mach. Theory.

[4] Awad L.N., Bae J., O’donnell K., De Rossi S.M., Hendron K., Sloot L.H., Kudzia P., Allen S., Holt K.G., Ellis T.D. (2017). A soft robotic exosuit improves walking in patients after stroke. Sci. Transl. Med..

[5] Morone G., Paolucci S., Cherubini A., De Angelis D., Venturiero V., Coiro P., Iosa M. (2017). Robot-assisted gait training for stroke patients: Current state of the art and perspectives of robotics. Neuropsychiatr. Dis. Treat..

[6] Au S., Berniker M., Herr H. (2008). Powered ankle-foot prosthesis to assist level-ground and stair-descent gaits. Neural Netw..

[7] Sup F., Varol H.A., Mitchell J., Withrow T.J., Goldfarb M. (2009). Preliminary evaluations of a self-contained anthropomorphic transfemoral prosthesis. IEEE ASME Trans. Mechatron..

[8] Narayan A., Reyes F.A., Ren M., Haoyong Y. (2021). Real-time hierarchical classification of time series data for locomotion mode detection. IEEE J. Biomed. Health Inform..

[9] Lee U.H., Bi J., Patel R., Fouhey D., Rouse E. (2020). Image transformation and CNNs: A strategy for encoding human locomotor intent for autonomous wearable robots. IEEE Robot. Autom. Lett..

[10] Varol H.A., Sup F., Goldfarb M. (2009). Multiclass real-time intent recognition of a powered lower limb prosthesis. IEEE Trans. Biomed. Eng..

[11] Hu B., Rouse E., Hargrove L. (2018). Fusion of bilateral lower-limb neuromechanical signals improves prediction of locomotor activities. Front. Robot. AI.

[12] Kazemimoghadam M., Fey N.P. (2022). An activity recognition framework for continuous monitoring of non-steady-state locomotion of individuals with Parkinson’s disease. Appl. Sci..

[13] Bloem B.R., Grimbergen Y.A., Cramer M., Willemsen M., Zwinderman A.H. (2001). Prospective assessment of falls in Parkinson’s disease. J. Neurol..

[14] Bloem B.R., Hausdorff J.M., Visser J.E., Giladi N. (2004). Falls and freezing of gait in Parkinson’s disease: A review of two interconnected, episodic phenomena. Mov. Disord. Off. J. Mov. Disord. Soc..

[15] Salarian A., Russmann H., Vingerhoets F.J., Burkhard P.R., Aminian K. (2007). Ambulatory monitoring of physical activities in patients with Parkinson’s disease. IEEE Trans. Biomed. Eng..

[16] Zwartjes D.G., Heida T., Van Vugt J.P., Geelen J.A., Veltink P.H. (2010). Ambulatory monitoring of activities and motor symptoms in Parkinson’s disease. IEEE Trans. Biomed. Eng..

[17] Huang H., Zhang F., Hargrove L.J., Dou Z., Rogers D.R., Englehart K.B. (2011). Continuous locomotion-mode identification for prosthetic legs based on neuromuscular–mechanical fusion. IEEE Trans. Biomed. Eng..

[18] Chen K., Zhang D., Yao L., Guo B., Yu Z., Liu Y. (2021). Deep learning for sensor-based human activity recognition: Overview, challenges, and opportunities. ACM Comput. Surv. CSUR.

[19] Ignatov A. (2018). Real-time human activity recognition from accelerometer data using convolutional neural networks. Appl. Soft Comput..

[20] Gupta S. (2021). Deep learning based human activity recognition (HAR) using wearable sensor data. Int. J. Inf. Manag. Data Insights.

[21] Lu L., Zhang C., Cao K., Deng T., Yang Q. (2022). A multichannel CNN-GRU model for human activity recognition. IEEE Access.

[22] Zhang C., Cao K., Lu L., Deng T. (2022). A multi-scale feature extraction fusion model for human activity recognition. Sci. Rep..

[23] Ismail Fawaz H., Forestier G., Weber J., Idoumghar L., Muller P.-A. (2019). Deep learning for time series classification: A review. Data Min. Knowl. Discov..

[24] Dang L.M., Min K., Wang H., Piran M.J., Lee C.H., Moon H. (2020). Sensor-based and vision-based human activity recognition: A comprehensive survey. Pattern Recognit..

[25] Szegedy C., Liu W., Jia Y., Sermanet P., Reed S., Anguelov D., Erhan D., Vanhoucke V., Rabinovich A. Going deeper with convolutions. Proceedings of the IEEE Conference on Computer Vision and Pattern Recognition.

[26] Tan T.-H., Gochoo M., Huang S.-C., Liu Y.-H., Liu S.-H., Huang Y.-F. (2018). Multi-resident activity recognition in a smart home using RGB activity image and DCNN. IEEE Sens. J..

[27] Ijjina E.P., Chalavadi K.M. (2017). Human action recognition in RGB-D videos using motion sequence information and deep learning. Pattern Recognit..

[28] Bahdanau D., Cho K., Bengio Y. (2014). Neural machine translation by jointly learning to align and translate. arXiv.

[29] Le Q., Mikolov T. Distributed representations of sentences and documents. Proceedings of the International Conference on Machine Learning.

[30] Sutskever I., Vinyals O., Le Q.V. Sequence to sequence learning with neural networks. Proceedings of the 27th International Conference on Neural Information Processing Systems.

[31] Goldberg Y. (2016). A primer on neural network models for natural language processing. J. Artif. Intell. Res..

[32] Young T., Hazarika D., Poria S., Cambria E. (2018). Recent trends in deep learning based natural language processing. IEEE Comput. Intell. Mag..

[33] Ordóñez F.J., Roggen D. (2016). Deep convolutional and LSTM recurrent neural networks for multimodal wearable activity recognition. Sensors.

[34] Xia K., Huang J., Wang H. (2020). LSTM-CNN architecture for human activity recognition. IEEE Access.

[35] Jain R., Semwal V.B., Kaushik P. (2022). Deep ensemble learning approach for lower extremity activities recognition using wearable sensors. Expert Syst..

[36] Khan I.U., Afzal S., Lee J.W. (2022). Human activity recognition via hybrid deep learning based model. Sensors.

[37] Dua N., Singh S.N., Semwal V.B. (2021). Multi-input CNN-GRU based human activity recognition using wearable sensors. Computing.

[38] Khan Z.N., Ahmad J. (2021). Attention induced multi-head convolutional neural network for human activity recognition. Appl. Soft Comput..

[39] Ronao C.A., Cho S.-B. (2016). Human activity recognition with smartphone sensors using deep learning neural networks. Expert Syst. Appl..

[40] Anguita D., Ghio A., Oneto L., Parra X., Reyes-Ortiz J.L. Human activity recognition on smartphones using a multiclass hardware-friendly support vector machine. Proceedings of the 4th International Workshop of Ambient Assisted Living and Home Care (IWAAL 2012).

[41] Roggen D., Calatroni A., Rossi M., Holleczek T., Förster K., Tröster G., Lukowicz P., Bannach D., Pirkl G., Ferscha A. Collecting complex activity datasets in highly rich networked sensor environments. Proceedings of the 7th International Conference on Networked Sensing Systems (INSS).

[42] Zappi P., Lombriser C., Stiefmeier T., Farella E., Roggen D., Benini L., Tröster G. Activity recognition from on-body sensors: Accuracy-power trade-off by dynamic sensor selection. Proceedings of the 5th European Conference of Wireless Sensor Networks (EWSN 2008).

[43] Yang J., Nguyen M.N., San P.P., Li X., Krishnaswamy S. Deep convolutional neural networks on multichannel time series for human activity recognition. Proceedings of the 24th International Joint Conference on Artificial Intelligence (IJCAI 2015).

[44] Kwapisz J.R., Weiss G.M., Moore S.A. (2011). Activity recognition using cell phone accelerometers. ACM SIGKDD Explor. Newsl..

[45] Weiss G.M., Yoneda K., Hayajneh T. (2019). Smartphone and smartwatch-based biometrics using activities of daily living. IEEE Access.

[46] Zhu L., Wang Z., Ning Z., Zhang Y., Liu Y., Cao W., Wu X., Chen C. (2020). A novel motion intention recognition approach for soft exoskeleton via IMU. Electronics.

[47] HEXAR-Humancare Hector H30A. https://hexarhc.com/?page_id=5465&lang=en.

[48] DELSYS Trigno Wireless Biofeedback System—User’s Guide. https://delsys.com/support/documentation/.

[49] Moreira L., Figueiredo J., Fonseca P., Vilas-Boas J.P., Santos C.P. (2021). Lower limb kinematic, kinetic, and EMG data from young healthy humans during walking at controlled speeds. Sci. Data.

[50] MotionAnalysis Kestrel-2200. https://www.motionanalysis.com/cameras/kestrel-2200/.

[51] He K., Sun J. Convolutional neural networks at constrained time cost. Proceedings of the IEEE Conference on Computer Vision and Pattern Recognition.

[52] Srivastava R.K., Greff K., Schmidhuber J. (2015). Highway networks. arXiv.

[53] He K., Zhang X., Ren S., Sun J. Deep residual learning for image recognition. Proceedings of the IEEE Conference on Computer Vision and Pattern Recognition.

[54] Li L., Jamieson K., DeSalvo G., Rostamizadeh A., Talwalkar A. (2017). Hyperband: A novel bandit-based approach to hyperparameter optimization. J. Mach. Learn. Res..

[55] Bergstra J., Bardenet R., Bengio Y., Kégl B. Algorithms for hyper-parameter optimization. Proceedings of the 24th International Conference of Neural Information Processing Systems, NIPS 2011.

[56] Bergstra J., Yamins D., Cox D. Making a science of model search: Hyperparameter optimization in hundreds of dimensions for vision architectures. Proceedings of the 30th International Conference on Machine Learning.

[57] Arora N., Mior M. Hyperopt: Distributed Hyperparameter Optimization. https://github.com/hyperopt/hyperopt.

[58] AI-Hub Motion Data of Walking Assistive Wearable Robot. https://aihub.or.kr/aihubdata/data/view.do?currMenu=115&topMenu=100&aihubDataSe=realm&dataSetSn=71526.

[59] Dehghani A., Sarbishei O., Glatard T., Shihab E. (2019). A quantitative comparison of overlapping and non-overlapping sliding windows for human activity recognition using inertial sensors. Sensors.

